# Supplementary material for: Conformational change of Syntaxin-3b in regulating SNARE complex assembly in the ribbon synapses
Source: Sci Rep. 2022 Jun 3;12:9261. doi: 10.1038/s41598-022-09654-3 (PMC9166750; doi:10.1038/s41598-022-09654-3)
Supplement: Supplementary file 1 — Supplementary Information 1. [file 41598_2022_9654_MOESM1_ESM.pdf]

Supplemental Figure 1

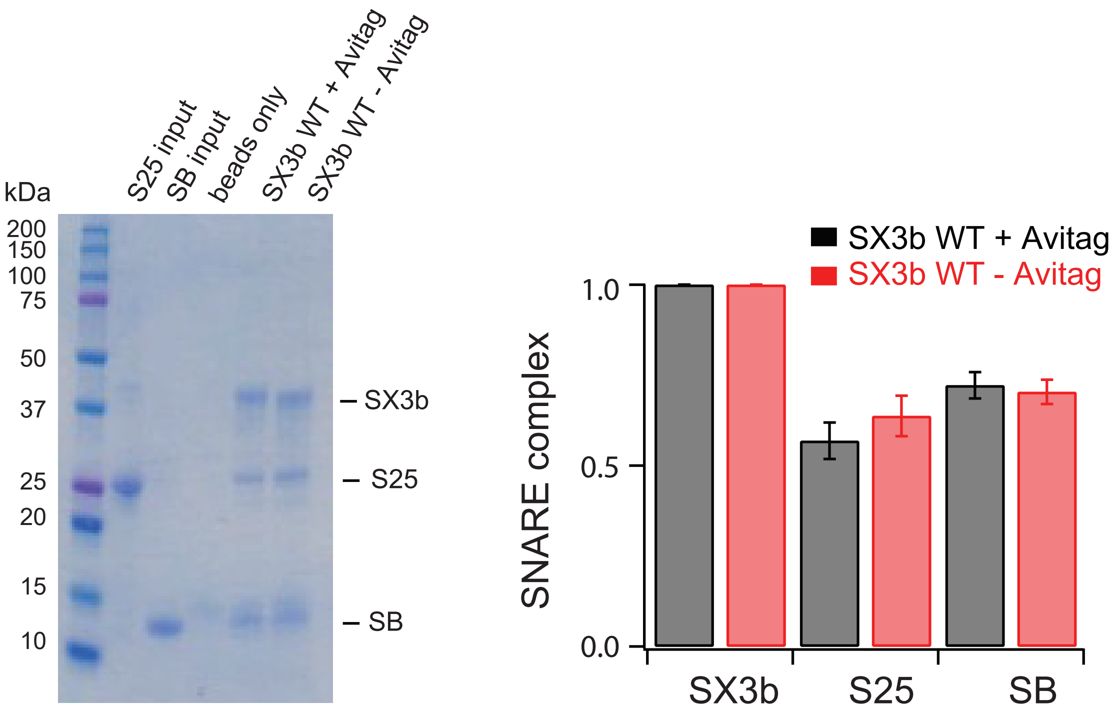

Supplemental Figure 1. The Avi-tag sequence does not affect ternary SNARE complex formation. A pulldown assay was conducted using biotinylated syntaxin-3b as bait on neutravidin coated beads to test the capability of ternary SNARE complex formation with SNAP-25 (S25) and synaptobrevin-2 (SB) in the presence and absence of Avi-tag sequence at the C-terminus of syntaxin-3b. Proteins bound to the beads were confirmed by SDS-PAGE. Syntaxin-3b, SNAP-25, synaptobrevin-2 bands were analyzed using ImageJ software (NIH, Bethesda, MD). Shown are means ± SD (n=3).
