# Supplementary material for: Conformational change of Syntaxin-3b in regulating SNARE complex assembly in the ribbon synapses
Source: Sci Rep. 2022 Jun 3;12:9261. doi: 10.1038/s41598-022-09654-3 (PMC9166750; doi:10.1038/s41598-022-09654-3)
Supplement: Supplementary file 2 — Supplementary Information 2. [file 41598_2022_9654_MOESM2_ESM.pdf]

Supplemental Figure 2

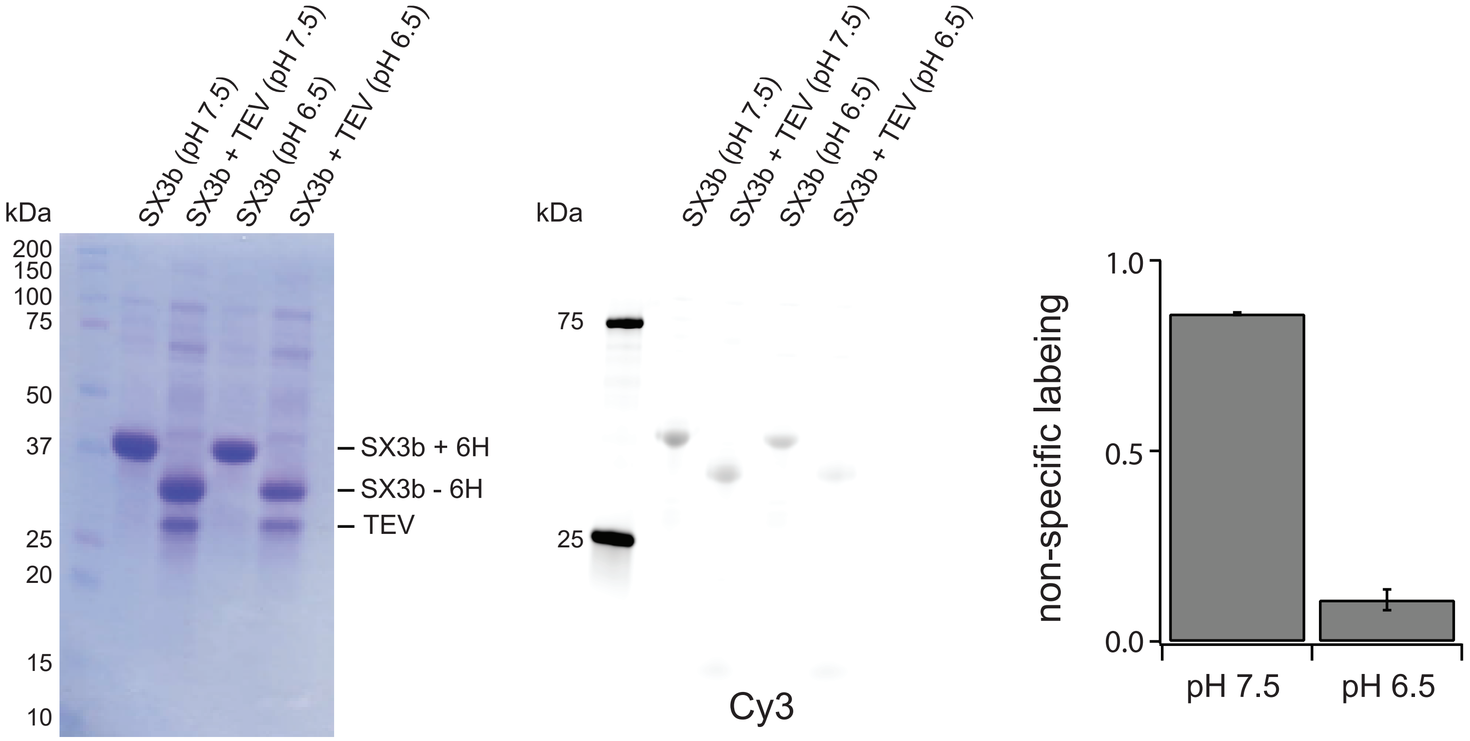

Supplemental Figure 2. Specific labeling at the N-terminal amino group using N-hydroxysuccinimide (NHS) ester. To validate the efficiency of N-terminal labeling using NHS ester, we labeled the syntaxin-3b construct consisting of an N-terminal 6x-histidine tag followed by a TEV protease cleavage site with Alexa555 NHS ester (ThermoFisher Scientific, Waltham, MA) at different pH, i.e., 6.5 and 7.5. When properly labeled at the N-terminal primary amino group, TEV protease would remove the fluorescence detection from syntaxin-3b. The samples were quantified by SDS-PAGE and imaged on the Typhoon to visualize the labeled samples prior to staining for protein levels. Syntaxin-3b bands before and after TEV cleavage were analyzed using ImageJ software (NIH, Bethesda, MD). Shown are means ± SD (n=3). Original Typhoon images are presented in Supplemental Figure 6.
