# Supplementary material for: Conformational change of Syntaxin-3b in regulating SNARE complex assembly in the ribbon synapses
Source: Sci Rep. 2022 Jun 3;12:9261. doi: 10.1038/s41598-022-09654-3 (PMC9166750; doi:10.1038/s41598-022-09654-3)
Supplement: Supplementary file 3 — Supplementary Information 3. [file 41598_2022_9654_MOESM3_ESM.pdf]

Supplemental Figure 3

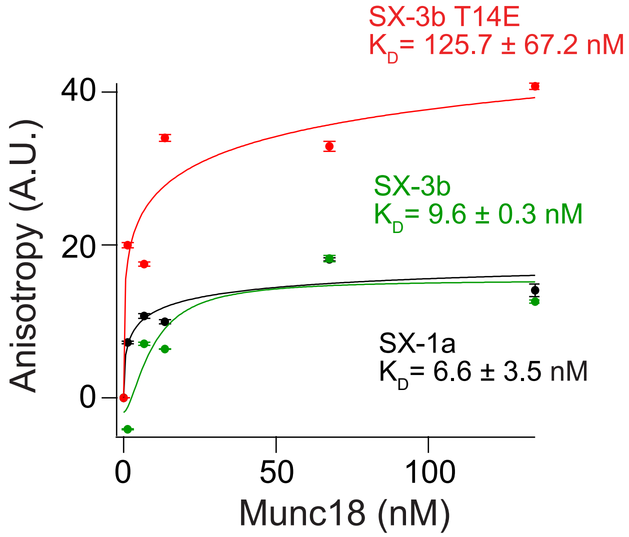

Supplemental Figure 3. Fluorescence anisotropy measurements. Bulk fluorescence anisotropy measurements of interactions between Alexa 488 labeled syntaxins and unlabeled Munc18 at 0 nM, 1.3 nM, 6.7 nM, 13.5 nM, 67.5 nM, 135 nM concentrations. The anisotropy curves are fit with Hill equations to estimate the disassociation constant K_d. Shown are means ± SD (n=3).
