# Supplementary material for: Conformational change of Syntaxin-3b in regulating SNARE complex assembly in the ribbon synapses
Source: Sci Rep. 2022 Jun 3;12:9261. doi: 10.1038/s41598-022-09654-3 (PMC9166750; doi:10.1038/s41598-022-09654-3)
Supplement: Supplementary file 4 — Supplementary Information 4. [file 41598_2022_9654_MOESM4_ESM.pdf]

Supplemental Figure 4

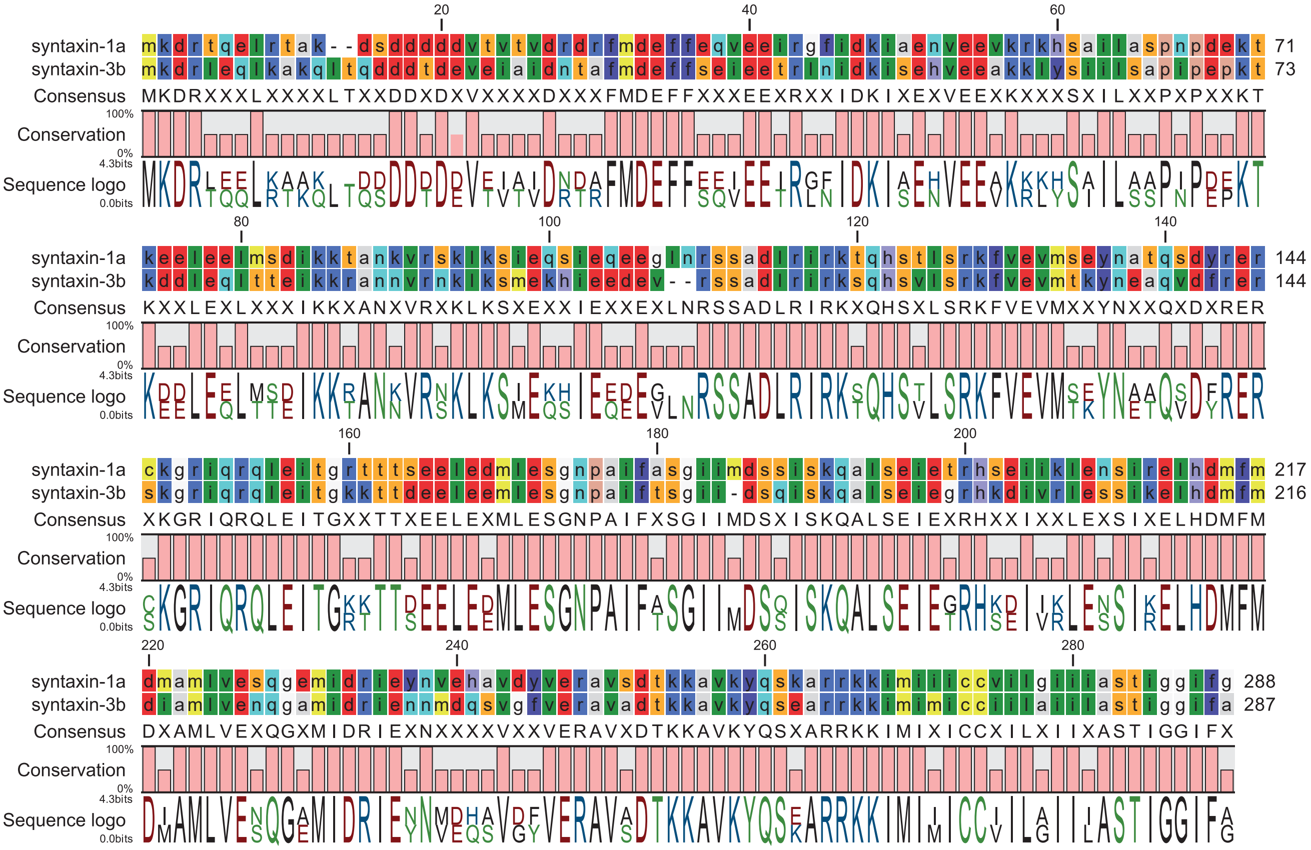

Supplemental Figure 4. Sequence alignment of syntaxin-1a and syntaxin-3b. The amino acid colors are based on the RasMol color scheme according to the properties of the amino acid, i.e., polar residues are bright colors and non-polar residues are dark colors.
