# Supplementary figures and images for: Conformational change of Syntaxin-3b in regulating SNARE complex assembly in the ribbon synapses
Source: Sci Rep. 2022 Jun 3;12:9261. doi: 10.1038/s41598-022-09654-3 (PMC9166750; doi:10.1038/s41598-022-09654-3)

Supplemental Figure 5

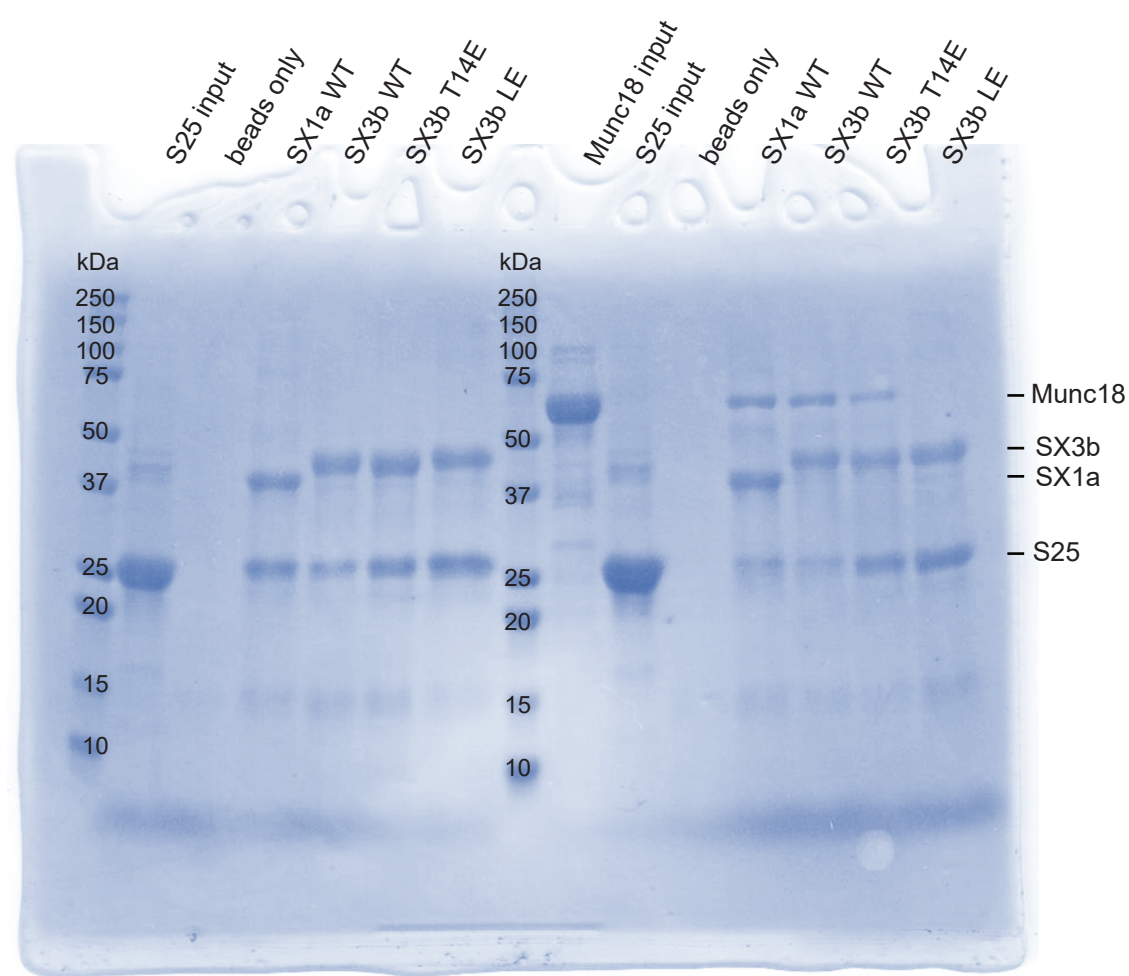

Supplemental Figure 5. Original SDS-PAGE gel from Figure 3F and 4F.

Supplement: Supplementary file 5 — Supplementary Information 5. [file 41598_2022_9654_MOESM5_ESM.pdf]
