# Supplementary material for: Conformational change of Syntaxin-3b in regulating SNARE complex assembly in the ribbon synapses
Source: Sci Rep. 2022 Jun 3;12:9261. doi: 10.1038/s41598-022-09654-3 (PMC9166750; doi:10.1038/s41598-022-09654-3)
Supplement: Supplementary file 6 — Supplementary Information 6. [file 41598_2022_9654_MOESM6_ESM.pdf]

Supplemental Figure 6

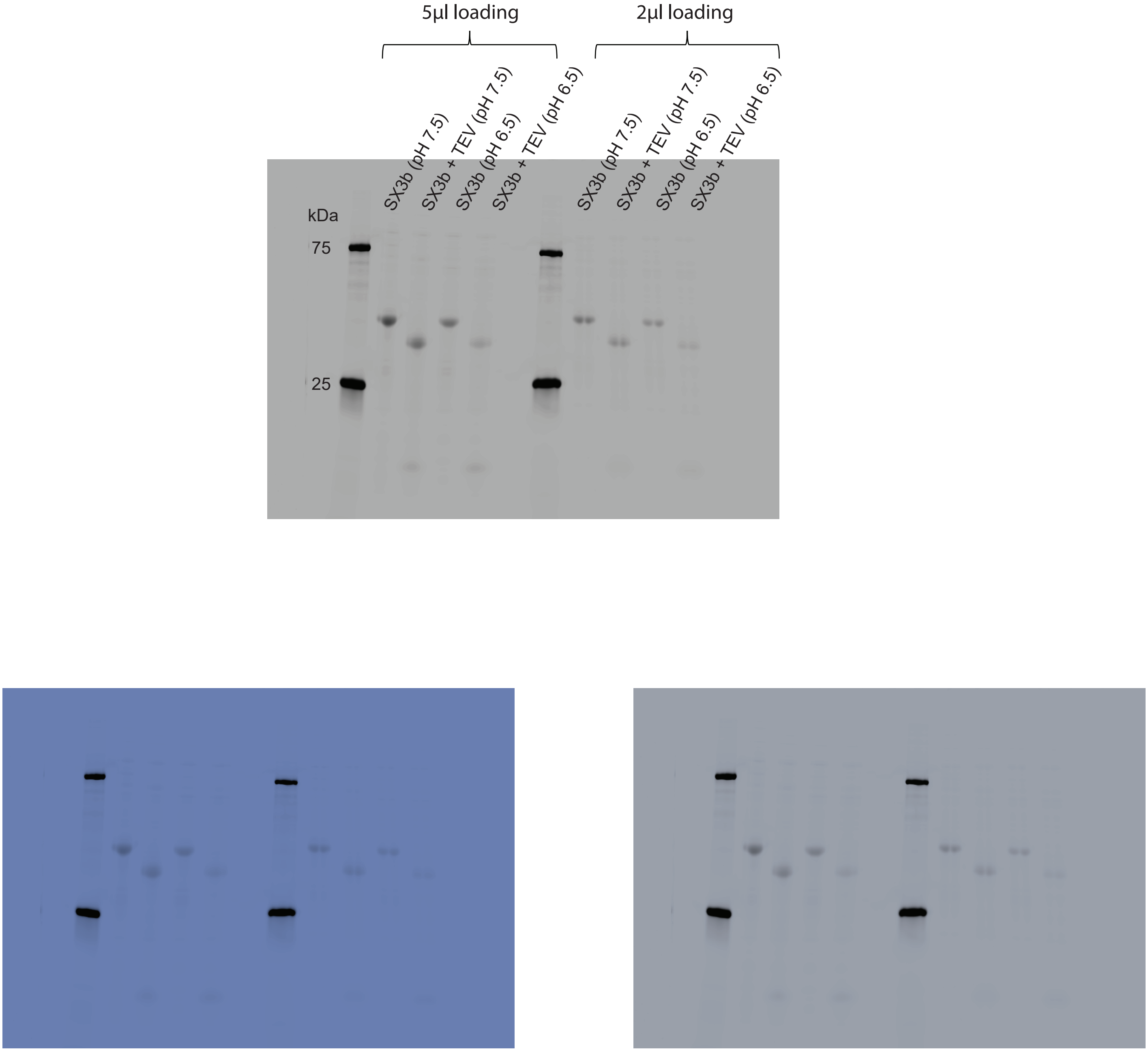

Supplemental Figure 6. Original Typhoon image of SDS-page gel. The SDS-page gel was imaged on the Typhoon to validate the specific N-terminal labeling using NHS ester at Cy3 excitation wavelength (~ 548nm).
